# Organic–Inorganic Nanostructure Architecture via Directly Capping Fullerenes onto Quantum Dots

**DOI:** 10.1007/s11671-010-9764-1

**Published:** 2010-09-02

**Authors:** Jae Kwan Lee, Jonggi Kim, Changduk Yang

**Affiliations:** 1 Research Center for Convergence Technology, Hoseo University, Chungnam 336-795, South Korea; 2 Interdisciplinary School of Green Energy, Ulsan National Institute of Science and Technology (UNIST), Ulsan 689-798, South Korea

**Keywords:** Quantum dots, Fullerene, Organic–inorganic nanostructure, Hybrid solar cell, Ligand exchange

## Abstract

A new form of fullerene-capped CdSe nanoparticles (**PCBA**-capped CdSe NPs), using carboxylate ligands with [60]fullerene capping groups that provides an effective synthetic methodology to attach fullerenes noncovalently to CdSe, is presented for usage in nanotechnology and photoelectric fields. Interestingly, either the internal charge transfer or the energy transfer in the hybrid material contributes to photoluminescence (PL) quenching of the CdSe moieties.

## Introduction

Buckminsterfullerenes, beside their structural attraction, have been extensively researched in materials science due to their unique properties such as the excellent electron affinity as well as the interesting photophysical/photochemical nature [1-5]. A parallel and equally heady progress has also been made toward the development of semiconductor nanoparticles (e.g., CdSe) since the variation of their particle size and shape provides continuous and predictable changes in optical/electronic properties and chemical processability [6-8]. In addition, these nanocrystals behave essentially as a three-dimensional potential well for electrons (i.e., the quantum mechanical 'particle in a box'). It thus comes as no surprise that both the fullerenes and the nanocrystals have been actively investigated for various applications; however, their fields have been largely independent so far and have enjoyed their own remarkable developments in the past [9-11]. For instance, both fullerenes and semiconductor nanocrystals have been blended with conjugated polymers respectively, giving rise to photovoltaic cells with high-power conversion efficiencies [12-22]. Despite given the prominent features of fullerenes and semiconductor nanocrystals, fullerene-based mixed nanocomposites of well-defined composition and stoichiometry have not been adequately studied [23-26]. These points have attracted our attention to chemically combine the two in a single unit so that '*synergy*' between the two can enhance and induce their outstanding properties.

To prepare the quantum dots (QDs)-based organic–inorganic hybrid nanomaterials, ligand exchange is a very useful method [27-29]. Even though thiol groups are the most used ligands for capping the QDs, they are unstable against photooxidation, which can lead to aggregation of the QDs [30-32]. Thus, we have turned to the carboxylic acid ligands for fullerene to CdSe nanocrystals. Recently, we reported the synthesis of the well-solubilized carboxylic acid fullerene (**PCBA**) (see below) for use as *n*-type materials in hybrid bulk-heterojunction solar cell [14].

**Figure 1:**
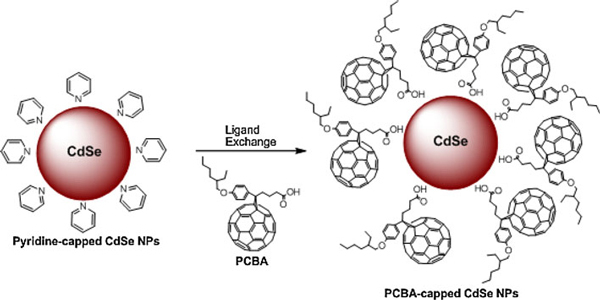
**Synthesis of PCBA-capped CdSe NPs**.

In the current work, through the ligand-exchange reaction, we describe here a route to prepare readily soluble nanocomposite of **PCBA**-CdSe (Figure 1). The structural and spectroscopic properties of the resulting **PCBA**-capped CdSe nanoparticles (NPs) are of great interest since such rational design and synthesis can lead potentially to promising protocol for use in optical and photoelectric fields. Either the internal charge transfer or the energy transfer phenomenon between CdSe core and **PCBA** shell is clearly observed, resulting in the photoluminescence (PL) quenching of CdSe.

## Experimental Section

### General

All solvents were purified and freshly distilled prior to use according to literature procedures. The synthesis of 4-(2-Ethylhexyloxy)-[6,6]-phenyl C_61_-butyric acid methyl ester (**PCBA**) [14], hexadecylamine-capped CdSe (HDA-capped CdSe) [18,33,34], and pyridine-capped CdSe (pyridine-capped CdSe) [34] was adapted from literature procedures. Commercially available materials were used as received unless noted.

### Ligand Exchange to Prepare PCBA-capped CdSe NPs

To the pyridine-capped CdSe nanocrystals (17 mg) dried with N_2_ gas, **PCBA** (66.5 mg, 0.065 mmol) was added along with 10 mL anhydrous THF. The mixture was then sonicated at room temperature for an hour while the nanoparticles gradually became fully soluble in the THF to form an optically clear solution. The exchange procedure was monitored by FT-IR (diminution of the carboxylic acid band at 1705 cm^-1^), which showed nearly quantitative formation after 3 h. The solution was then dried by bubbling Ar and then stored in glove box.

## Results and Discussion

(6,6)-Phenyl C_61_-butyric acid methyl ester (PCBM) is a soluble form of fullerenes as well as the most widely used organic *n*-type material. The PCBM can be hydrolyzed with a strong acid to the corresponding carboxylic acid PCMA which is a hardly soluble, not processable material. The introduction of a branched side chain as a solubilizer on the phenylene of PCBM (**PCBA**) [14] allows the excellent solubility, which enables to study the organic–inorganic hybrid material containing C_60_.

The hexadecylamine (HDA)-capped CdSe nanoparticles (CdSe NPs) were prepared using the reported procedures [18,33,34]. Then, HDA-capped CdSe NPs were successfully transformed to the corresponding pyridine-capped CdSe NPs by the treatment with pyridine under ultrasonication [34]. Subsequently, the ligand exchange with **PCBA** afforded the **PCBA**-capped CdSe NPs which is readily soluble in organic solvents (THF, chloroform, toluene, etc.) (Figure 1).

**Figure 2:**
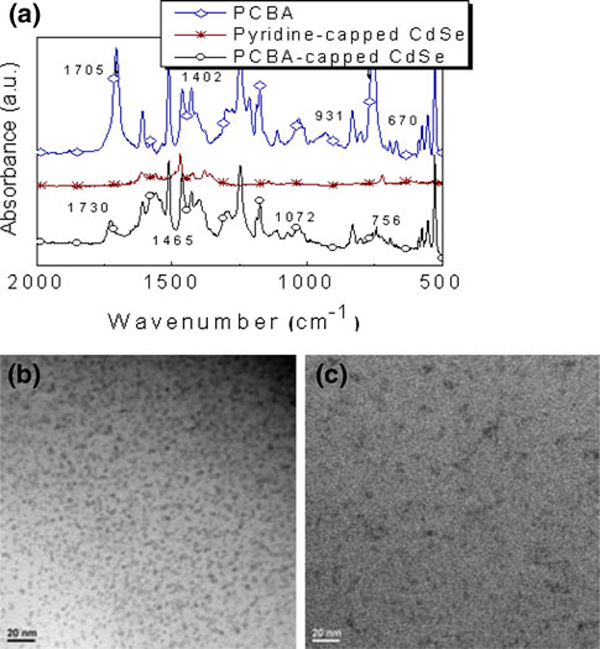
**a FT-IR spectra on KBr for dropcast samples of PCBA (*diamond*), CdSe NPs (*asterisk*), PCBA-CdSe NPs (*circle*); b TEM of pyridine-capped CdSe NPs and c PCBA-CdSe NPs**.

To ensure that the ligand exchanges had successfully gone to completion, the NPs were characterized by FT-IR (Figure 2). On a low-energy region (from 1,800 to 1,500 cm^-1^), the FT-IR spectrum of the pristine **PCBA**, displays a strong absorption band at 1,705 cm^-1^ assigned to the carboxylic acid, which is not present in the pyridine-capped CdSe NPs. In contrast, the **PCBA**-capped CdSe NPs reveal somewhat similar to the **PCBA**; however, it appears that the carboxylic acid band is diminished and shifted to lower energy at 1,730 cm^-1^ with a relatively decrease in intensity. The inspection of FT-IR of the **PCBA**-capped CdSe NPs such as the disappearance of the carboxylic acid band and new broad bands around 1,570 cm^-1^ is clearly suggestive that carboxylate binding are formed between **PCBA** and CdSe through the ligand exchange reaction.

Figure 2b and 2c depict a transmission electron microscopy (TEM) micrograph of the NPs (pyridine-capped CdSe and **PCBA**-capped CdSe). The TEM image of the pyridine-capped CdSe is characteristic of uniformly shaped spheres, with a mean size of 5.0 ± 1 nm (see Figure 2b). In the case of analyzing the **PCBA**-capped CdSe, the monodispersed spheres are not easily seen in the resolution TEM; however, a nanometer-sized pattern of larger objects is observed. This can be attributed to the nanoagglomerated feature, which is resulted from the interaction between the **PCBA** attached to the CdSe. In addition, we cannot rule out that more than two CdSe units can be surrounded by **PCBA** moieties, which probably leads to the roughly spherical form.

**Figure 3:**
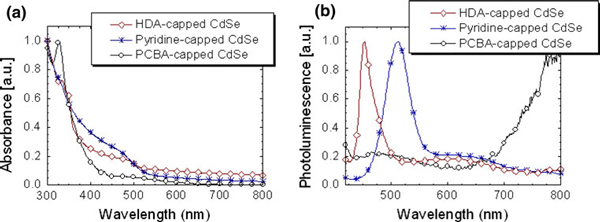
**a UV–Vis absorption and b PL spectra of HDA-capped CdSe NPs (*diamond*), pyridine-capped CdSe NPs (*asterisk*), and PCBA-capped CdSe NPs (*circle*) in chloroform solution**.

The UV–Vis and photoluminescence (PL) properties of all the NPs (HDA-capped CdSe, pyridine-capped CdSe and **PCBA**-capped CdSe) were investigated in chloroform solution. As shown in Figure 3, the solution optical spectra of both HDA-capped CdSe and pyridine-capped CdSe exhibit broad absorption bands (410–460 nm), arising from exciton absorption peak in CdSe NPs. In the case of **PCBA**-capped CdSe, the distinguishable band at 330 nm corresponds to the characteristic of C_60_ not only exhibits but also the absorption feature is almost identical to that of the pristine **PCBA**. This implies that **PCBA** is completely binding along the CdSe unit.

In PL spectrum of the pyridine-capped CdSe NPs, the bathochromic shift of the onset of the emission of ca. 60 nm is observed when compared to the HDA-capped CdSe NPs. This low-energy emission can be due to aggregated formation and increased intermolecular interaction in the NPs [35]. In the case of the **PCBA**-capped CdSe in solution, the PL quenching of the band at 512 nm corresponding to CdSe is clearly observed, and an emission peak centered at 795 nm which stems from the **PCBA** units becomes dominant. The decrease in PL intensity in CdSe NP is mostly likely caused by the quenching of photoexcited CdSe NP through charge transfer to the electron-accepting **PCBA** from CdSe NPs. However, we cannot rule out that, through utilization of the carboxylates as a linkage between CdSe with **PCBA**, it is possible to trigger the energy transfer in the hybrid material since the length scale of domain in **PCBA**-capped CdSe is smaller than typical values for Förster energy transfer (<10 nm) [36]. To confirm that the **PCBA**-capped CdSe of the intracomplex quenching is indeed charge injection of a conduction band electron of the photoexcited CdSe NPs to the surface-bound **PCBA**, we are complementing our studies by time-resolved transient absorption spectroscopic measurements with CdSe NPs and **PCBA**-capped CdSe.

Both PCBM and CdSe derivatives have been applied as the electron transport materials in the most widely used configuration of the photovoltaic cells [14,21,37-39]. Furthermore, the nearly complete quenching of emission from the higher energy CdSe NPs to **PCBA** may be a significant key in further improving the efficiency of an organic solar cell.

In addressing this fundamental question, we are currently studying the hybrid nanostructures fabricated with conjugated polymers, in that enables to create the positive aspect in the hybrid solar cells and other hybrid materials-based optical and electrooptical applications via the ligand exchange.

## Conclusion

In summary, we have demonstrated the preparation of soluble nanoparticle of **PCBA**-CdSe by directly exchanging the ligands onto the surface of pyridine-capped CdSe, which is fully characterized by FT-IR, UV–Vis absorption, PL spectra, and TEM. The resulting **PCBA**-capped CdSe in solution shows a dominant emission peak centered at 795 nm, which is originated from the **PCBA** units. It clearly indicates that either the internal charge transfer or the energy transfer from the CdSe to **PCBA** moieties in solution occurs, so that the emission band (λ_max_ = 512 nm) from the CdSe units is nearly quenched. We envision that a hybrid-polymeric nanoarchitecture can be utilized for constructing one layer of solar cell to explore the possible optoelectronic device application, which will open up new opportunities for the development of solar cells based on organic–inorganic nanocomposites. This work is currently under investigation.
